# In-Hospital Outcomes of Acute Myocarditis in Adults With Systemic Inflammatory Disease: A Perspective on the Nationally Representative Sample Within the United States

**DOI:** 10.7759/cureus.88079

**Published:** 2025-07-16

**Authors:** Olufemi P Odugbemi, Caroline Apreku, Samuel Obasi-Eze, Godfrey Tabowei, Ruth Pius

**Affiliations:** 1 Internal Medicine, Lincoln Medical and Mental Health Center, New York, USA; 2 Internal Medicine, New York-Presbyterian Brooklyn Methodist Hospital, New York, USA; 3 Internal Medicine, Alex Ekwueme Federal University Teaching Hospital, Abakaliki, NGA; 4 Internal Medicine, Texas Tech University Health Sciences Center, Odessa, USA

**Keywords:** acute myocarditis, heart failure, major adverse cardiac and cerebrovascular events (macce), ms (multiple sclerosis), psoriasis, rheumatoid arthritis, sle, systemic inflammatory disease, systemic lupus erythematous (sle), hashimoto’s thyroiditis

## Abstract

Background

Systemic inflammatory diseases (SID) are associated with adverse cardiovascular (CV) events due to the derangement in innate immunity. Understanding the association with acute myocarditis (AMC) is crucial for preemptive management strategies and improving patient outcomes.

Methods

We identified and compared adults (>18 years) with SID versus non-SID who were hospitalized with AMC using a large, nationally representative inpatient database from 2016 to 2021 and standardized diagnostic codes. Six SIDs were selected based on the distribution of human leukocyte antigens (HLA). The risks of all-cause mortality and major adverse cardiac and cerebrovascular events (ischemic stroke, cardiac arrest, acute heart failure (AHF), ventricular arrhythmia (VA), complete atrioventricular block, acute myocardial infarction (AMI)) and use of circulatory support were assessed in adult AMC admissions with and without SID after adjusting for confounders. SID was divided into six cohorts: systemic lupus erythematosus (SLE), rheumatoid arthritis (RA), psoriasis (PS), inflammatory bowel disease (IBD), Hashimoto's thyroiditis, and multiple sclerosis (MS) subgroups.

Results

Overall, there were 34,540 adult admissions to AMC. Prevalence of AMC in non-SID 96% (34,540/35,865). The mean age of non-SID was similar across the four cohorts, but relatively older in RA and MS (60.8 and 59.5 years, respectively). The cohorts also had a higher proportion of females in the PS and IBD cohorts.

Hypertension, peripheral vascular disease, and smoking were more frequently seen in the SID-AMC cohort. At the same time, obesity was more regularly seen in RA and MS, diabetes mellitus (DM) was more commonly seen in SLE and MS, and hyperlipidemia was more frequent with SLE, RA, PS, and MS.

The RA-AMC cohort had significantly higher adjusted odds of using mechanical circulatory support (adjusted odds ratio (aOR) 2.8, CI 1.22-6.55, p = 0.015) after adjusting for sex, dyslipidemia, DM, and chronic kidney disease (CKD).

However, odds outcomes of acute, VA, mortality, and major adverse cardiovascular events were not statistically significant.

Conclusions

The RA-AMC cohort is 280% more likely to require mechanical circulatory support during hospitalization for AMC, suggesting worse AHF and cardiogenic shock outcomes. Prospective studies would be necessary to further examine the cardiovascular outcomes in this cohort.

## Introduction

The myocardium, the heart’s thick middle layer, comprises specialized cardiac myocytes that generate the contractile force essential for sustaining life. Positioned between the endocardium internally and the epicardium externally, this muscular tissue is susceptible to a variety of insults, such as infectious agents, pharmacologic toxins, or dysregulated immune responses, that provoke inflammation, collectively termed myocarditis [1,2].Clinically, the manifestation of myocarditis spans a spectrum from subtle, subclinical injury to fulminant heart failure and sudden cardiac death. Although frequently underrecognized, hospital data from England demonstrate a nearly 90% rise in admissions for primary myocarditis over the past twenty years, most commonly affecting young adults, particularly men, and with a median hospitalization of just over four days [3].

Myocarditis is classified based on clinical course and severity. By duration, it is termed acute when symptom onset to diagnosis occurs within one month, subacute when spanning one to three months, and chronic when persisting beyond three months [3]. Severity ranges from mild, self-limited presentations to fulminant forms that necessitate inotropic support or mechanical circulatory assistance [4]. Histopathologically, myocarditis is characterized by an inflammatory cellular infiltrate, either focal or diffuse, accompanied by variable degrees of myocyte injury [5]. The nature of the infiltrate is related to the key subtypes: lymphocytic myocarditis, frequently post-viral or autoimmune; eosinophilic myocarditis, linked to hypersensitivity reactions or parasitic infestations; giant-cell myocarditis, a severe idiopathic variant occasionally seen alongside systemic autoimmune disorders; and granulomatous myocarditis, most commonly associated with sarcoidosis.

Over time, definitive diagnosis of suspected acute myocarditis (AMC) relied heavily on endomyocardial biopsy to demonstrate inflammatory infiltrates and fibrosis [6].However, because of the patchy distribution of infiltrates and the procedure carries substantial risks outside expert centers, its practical benefit remains limited. The advent of high-sensitivity troponin assays and cardiac magnetic resonance (CMR) imaging has transformed diagnostic practice, enabling accurate, non-invasive detection of myocardial injury and tissue characterization across a broader patient population [7].Despite these advances, CMR cannot reliably distinguish histologic subtypes, rendering biopsy indispensable in selected high-risk scenarios to guide targeted therapeutic interventions.

Systemic inflammatory diseases (SIDs) carry cardiovascular risks, which may extend beyond isolated myocardial injury. Chronic immune-mediated disorders, such as rheumatoid arthritis, systemic lupus erythematosus, and inflammatory bowel disease, are associated not only with accelerated atherosclerosis and an elevated incidence of myocardial infarction but also with inflammatory cardiomyopathies and myocarditis [8]. Lupus or sarcoidosis can directly target myocardial tissue through dysregulated innate and adaptive immunity [9]. Comprehensive evaluation, including advanced imaging modalities, targeted biopsy when indicated, and extracardiac assessment, is essential to distinguish primary infectious myocarditis from myocarditis driven by systemic inflammation.

In critical care settings, AMC often presents with electrical instability such as high-grade atrioventricular block, ventricular arrhythmias, or with acute heart failure. Cardiac sarcoidosis and giant-cell myocarditis exemplify the overlap between systemic inflammation and fulminant myocardial injury: although they may present similarly, their management diverges markedly. Cardiac sarcoidosis typically requires combined device therapy (pacemaker-defibrillator implantation) alongside immunomodulatory treatment, whereas giant-cell myocarditis demands early, aggressive immunosuppression to improve survival [10,11]. Early recognition of these high-risk forms of myocarditis and a diagnostic and therapeutic approach tailored to the underlying etiology are paramount. By integrating clinical presentation, non-invasive imaging, and histopathological confirmation when necessary, clinicians can optimize outcomes for patients whose myocardial inflammation reflects a broader systemic process, ensuring that therapy addresses both cardiac injury and its systemic drivers.

In this study, we aim to explore whether adults admitted with AMC who have coexisting systemic inflammatory diseases experience different in-hospital outcomes, including mortality, cardiogenic shock, acute heart failure, arrhythmias, need for mechanical circulatory support or coronary intervention, and length of stay. By comparing these outcomes across individual SID subtypes, we aim to reveal how distinct inflammatory diseases influence the clinical trajectory of myocarditis and inform more personalized patient care.

Viruses have long been implicated in AMC, yet their pathogenic role extends far beyond direct cytotoxicity. After initial infection by parvovirus B19, adenovirus, or human herpesvirus-6, viral particles may adhere to endothelial or myocyte membranes, triggering an innate immune response marked by cytokine release and activation of antigen-presenting cells [12]. Pattern recognition receptors detect viral components, inducing interferon and interleukin production that amplifies local inflammation. Although viral genomes are often recovered from myocardial tissue, their mere presence does not predict inflammation or prognosis; rather, a dysregulated immune reaction, driven by cytokine storms or molecular mimicry between viral antigens and cardiac proteins, emerges as the principal mediator of myocyte injury and necrosis [13]. In influenza-associated myocarditis, where viral RNA may remain confined to the respiratory tract, fulminant myocardial inflammation can ensue, illustrating that remote respiratory infection may precipitate life-threatening cardiac injury [14].

Under normal conditions, peripheral tolerance mechanisms, including the immune checkpoints CTLA-4 (cytotoxic T-lymphocyte antigen 4) and PD-1 (programmed cell death protein 1), prevent self-reactive T cells from attacking healthy tissues [15]. These receptors act as “brakes,” down-regulating T-cell activation when they engage their ligands on antigen-presenting cells or peripheral tissues. If the function of these receptors is impaired, autoreactive cytotoxic T-cell clones can expand unchecked, recognize shared epitopes on cardiac myocytes, and infiltrate the myocardium, leading to myocyte injury and necrosis [16]. Immune-checkpoint inhibitors (also called checkpoint blockade therapies) block CTLA-4 or PD-1 to unleash T cells against cancer [17]. While this enhances antitumor immunity, it simultaneously disrupts peripheral tolerance to T cells. The results in T-cell overactivation can target cardiac tissue, causing iatrogenic myocarditis. This form of myocarditis typically manifests within weeks of therapy initiation and is characterized by dense, aggressive T-cell infiltrates [17]. The cardiotoxic effects of these inhibitors carry a high mortality, especially in older patients with comorbidities. The mechanism of action of these inhibitors underscores the role of the delicate balance between mounting a robust antitumor response and maintaining self-tolerance in the pathogenesis of myocarditis.

Although bacterial myocarditis is rare, it exemplifies direct microbial invasion and toxin-mediated injury. Bacterial organisms such as *Staphylococcus aureus* and *Streptococcus pneumoniae* can penetrate the myocardial interstitium, provoking neutrophil-predominant inflammation and microabscess formation [18]. In these cases, pathogenesis is driven by pathogens that recruit innate immune cells and trigger endothelial activation; without prompt antimicrobial therapy, this inflammatory cascade inevitably progresses to tissue necrosis and fibrosis.

Autoimmune myocarditis adds yet another layer, arising from intrinsic immune dysregulation. Genetic predispositions affecting major histocompatibility complex (MHC) and non-MHC loci skew antigen presentation, while an imbalance between Th17 effector cells and regulatory T cells amplifies pro-inflammatory signalling [19]. Exposure to intracellular cardiac antigens or molecular mimicry may stimulate autoantibody production against myosin, troponin, or adrenergic receptors, perpetuating myocardial damage even after pathogen clearance [20]. The pathogenesis of autoimmunity in myocarditis is demonstrated in Giant-cell myocarditis, where multinucleated giant cells and T-cell infiltrates drive rapid myocardial destruction [21]. In contrast, eosinophilic myocarditis reflects toxic granule release by eosinophils under the influence of interleukin-5 and related cytokines.

The manifestations of SIDs are usually multisystemic, affecting the cardiovascular system. In sarcoidosis, non-necrotizing granulomas arise from maladaptive macrophage-T-cell interactions targeting myocardial tissue, commonly the basal septum, disrupting conduction and precipitating arrhythmias [22]. Systemic sclerosis can invoke a cardiac Raynaud’s phenomenon, where vasospasm-induced ischemia and persistent transforming growth factorβ signaling drive fibroblast activation, extracellular matrix deposition, and diastolic dysfunction [23]. Lupus myocarditis combines immune complex deposition, complement activation, and anti-heart autoantibodies, yielding patchy lymphocyte-rich infiltrates detectible only by advanced imaging [24]. Even extracardiac diseases such as psoriasis can provoke myocarditis through systemic interleukin-17A-driven inflammation and cross-reactive autoantibodies, demonstrating that chronic skin immune activation may affect the heart [25].

Despite this diversity of triggers, these pathogenic routes converge on a final common pathway: inflammation-induced myocyte death, extracellular matrix remodelling, and, if unchecked, progression to dilated cardiomyopathy. Recognizing the specific immunopathogenic processes at work, whether due to systemic inflammatory diseases, virus-triggered cytokine storms, immune-checkpoint inhibitor-induced T-cell activation, or autoantibody-mediated injury, is essential.

## Materials and methods

Data source 

We conducted this study using the 2016-2021 National Inpatient Sample (NIS) maintained by the Agency for Healthcare Research and Quality (AHRQ) under the Healthcare Cost and Utilization Project (HCUP). The NIS compiles inpatient discharge information from various non-federal, short-term, acute care hospitals across the United States, employing a stratified probability sampling framework. Sampling strata are defined by characteristics such as geographic region, rural or urban designation, hospital bed size, ownership type, and teaching status. Within each stratum, 20% of hospitals are randomly chosen, and their data are then weighted to provide nationally representative estimates. Because the data are fully de-identified and publicly available, the research did not require Institutional Review Board approval or consent.

Study design and population

This study was a cross-sectional analysis using inpatient data from the 2016-2021 NIS. From an initial pool of approximately 208 million hospitalization involving patients aged 18 years or older, we identified individuals with a documented diagnosis of acute myocarditis through the International Classification of Diseases, 10th Revision, Clinical Modification (ICD-10-CM) [26] codes "I409", "I400", "I401", and "I408", with either of the six systemic inflammatory diseases (SID) selected based on their human leucocyte antigen (HLA). These were identified using their ICD-10-CM codes as follows; systemic lupus erythematosus (SLE) ("M329"), rheumatoid arthritis (RA) ("M0600", "M069", and "M0680"), psoriasis (PS) ("L409", and "L4050"), multiple sclerosis (MS) ("G35"), inflammatory bowel disease (IBD) ("K50", and "K51"), and Hashimoto thyroiditis (HT) ("E063").

Outcomes

Our primary outcomes including major cardiovascular events including, ventricular arrhythmia ("I470", "I472", "I4901", "I4902"), acute heart failure ("I5021", "I5023", "I5031", "I5033", "I5041", "I5043"), cardiogenic shock ("R570"), acute myocardial infarction ("I219", "I2101", "I2102", "I2109", "I2111", "I2119", "I2121", "I2129", "I213", "I214"), and cardiac arrest ("I46," "I462," "I468," "I469") were compared amongst patients with SID and without SID. Covariates were identified using the corresponding ICD-10-CM codes. A complete list of codes can be found in the supplementary material.

Statistical analysis

The patient's demographics and comorbidities were compiled to summarize the baseline characteristics, presenting means (with standard deviations) for continuous variables and proportions for categorical variables. Given the extensive sample size, comparisons of categorical data were performed using Pearson's chi-square tests, whereas continuous variables were assessed using Student's t-tests (relying on the assumptions of the central limit theorem).

We first calculated unadjusted odds ratios (ORs) using univariate logistic regression, along with 95% confidence intervals (CIs) and p-values, to explore the risk association between acute myocarditis and each SID. We then created a multivariable logistic regression model to account for potential confounders. The adjusted analysis included the following covariates: age, gender, pre-existing heart failure, prior myocardial infarction, diabetes mellitus, hypertension, chronic kidney disease, smoking status, and chronic kidney disease.

All statistical procedures were performed in Stata® version 18 (StataCorp LP, College Station, TX). We used the survey ("svy") commands to incorporate NIS-specific sampling weights and stratification parameters. Statistical significance was defined at a two-sided p-value < 0.05.

## Results

The selection process for identifying hospitalizations with AMC in the database. Out of 208,109,925 total hospitalizations in the specified period, 35,865 cases were identified with AMC. Of these, 1,325 (3.7%) involved patients with six specified SID, while the remaining 34,540 (96.3%) had no documented SID.

From Table 1, among patients hospitalized primarily for acute myocarditis, those with co-existing systemic inflammatory diseases demonstrated demographic patterns that were different when compared to patients without SID. Patients with rheumatoid arthritis and multiple sclerosis tended to be older than those without SID, while the others showed age distributions more similar to the general population. Sex distribution varied across groups. Female patients were predominant in conditions like psoriasis and IBD, whereas they were underrepresented in groups such as SLE and rheumatoid arthritis.

**Table 1:** Characteristics of study population SLE: systemic lupus erythematosus; RA: rheumatoid arthritis; PS: psoriasis; IBD: inflammatory bowel disease; HT: Hashimoto thyroiditis; MS: multiple sclerosis; COPD: chronic obstructive pulmonary disease; SID: systemic inflammatory disease *P-value <0.05 considered significant

Variables	Systemic inflammatory disease (1,325)						No-SID (34,540)	P-value	Pearson Chi-square
-	SLE (235)	RA (375)	PS (205)	IBD (250)	HT (120)	MS (140)	-	-	-
Age, mean (SD)	47.6 (15.9)	60.8 (15.9)	45.8 (18.7)	47.6 (19.2)	45.3 (13.9)	59.5 (12.1)	48 (19.5)	<0.001*	-
Female, N (%)	31 (13%)	75 (20%)	115 (56%)	150 (60%)	40 (33%)	20 (14%)	21,414 (62%)	<0.001*	7.91
Comorbidities									
Obesity, N (%)	49 (21%)	75 (20%)	31 (15%)	45 (18%)	16 (13%)	35 (25%)	6,563 (19%)	<0.001*	1.2
Chronic kidney disease, N (%)	13 (31%)	41 (11%)	25 (12%)	30 (12%)	5 (4%)	10 (7%)	3,799 (11%)	<0.001*	2.07
Diabetes, N (%)	9 (21%)	34 (9%)	10 (5%)	20 (8%)	0	29 (21%)	2,072 (6%)	<0.001*	3.93
Hyperlipidemia, N (%)	66 (28%)	180 (48%)	76 (37%)	65 (26%)	30 (25%)	60 (43%)	9,671 (28%)	<0.001*	1.13
Hypertension, N (%)	49 (21%)	131 (35%)	41 (20%)	36 (15%)	22 (18%)	18 (13%)	11,053 (32%)	<0.001*	1.07
Tobacco, N (%)	32 (15%)	135 (36%)	35 (17%)	45 (18%)	20 (17%)	45 (32%)	5,872 (17%)	<0.001*	1.03
Prior myocardial infarction, N (%)	5 (2%)	19 (5%)	14 (7%)	5 (2%)	0	0	1,727 (5%)	<0.001*	2.27
Obstructive sleep apnea, N (%)	31 (13%)	34 (9%)	0	15 (6%)	5 (4%)	6 (4%)	2,418 (7%)	<0.001*	1.75
Peripheral vascular disease, N (%)	5 (2%)	15 (4%)	14 (7%)	0	0	0	345 (1%)	<0.001*	1.15
Heart failure, N (%)	110 (47%)	180 (48%)	84 (41%)	85 (34%)	55 (46%)	70 (50%)	13816 (40%)	<0.001*	4.47
COPD, N (%)	9 (4%)	79 (21%)	31 (15%)	20 (8%)	0	10 (7%)	2,763 (8%)	<0.001*	1.44
Hospital region, N (%)									
Northeast, N (%)	45 (19%)	79 (21%)	25 (12%)	45 (18%)	40 (33%)	50 (36%)	7,599 (22%)	< 0.001*	9.5
Midwest, N (%)	59 (25%)	98 (26%)	49 (24%)	55 (22%)	25 (21%)	20 (14%)	7,944 (23%)	< 0.001*	9.5

There were also notable racial and ethnic differences, with Caucasians more likely to have multiple sclerosis and Hashimoto thyroiditis. In contrast, Black patients were more frequently represented among those with SLE and underrepresented in other SID groups.

Differences in lifestyle and comorbid conditions were also evident. Smoking was more commonly reported in rheumatoid arthritis and multiple sclerosis; cardiovascular comorbidities were more frequent among patients with SIDs than in the no-SID group.

Patients with SID experienced a higher rate of acute heart failure (31.3% vs. 27.5%) and complete heart block (2.9% vs. 2.1%) (Table 2). Acute ischemic stroke was slightly more common among SID patients (2.4% vs. 2%), and so was cardiac arrest (4.2% vs. 3.6%). Other health complications were largely similar between groups.

**Table 2:** Proportion of clinical outcomes SID vs. no-SID SID: systemic inflammatory disease

Clinical events	SID	No-SID
In-hospital mortality, N (%)	109 (8.2%)	3,074 (8.9%)
Ventricular arrhythmias, N (%)	127 (9.6%)	3,868 (11.2%)
Cardiogenic shock, N (%)	171 (12.9%)	4,801 (13.9%)
Acute heart failure, N (%)	415 (31.3%)	9,498 (27.5%)
Acute ischemic stroke, N (%)	32 (2.4%)	691 (2%)
Complete heart block, N (%)	38 (2.9%)	725 (2.1%)
Acute respiratory failure, N (%)	350 (26.4%)	9,222 (26.7%)
Acute kidney injury, N (%)	368 (27.8%)	10,224 (29.6%)
Cardiac arrest, N (%)	56 (4.2%)	1,243 (3.6%)
Mechanical circulatory support, N (%)	70 (5.3%)	1,762 (5.1%)
Length of stay in days (mean)	8.6	7.6
Total hospital charge in $ (mean)	139423.5	148133.7

Acute respiratory failure, acute kidney injury (AKI), and cardiac arrest were highest in MS patients than in other groups (Table 3). Correspondingly, mean total hospital charges were highest in IBD ($211,938.3), followed by HT and MS ($171,750.6 each). Acute respiratory failure, acute kidney injury (AKI), and cardiac arrest were highest in MS patients than in other groups. Correspondingly, mean total hospital charges were highest in IBD ($211,938.3), followed by HT and MS ($171,750.6 each).

**Table 3:** Proportion of clinical outcomes within each SID cohort SLE: systemic lupus erythematosus; RA: rheumatoid arthritis; PS: psoriasis; IBD: inflammatory bowel disease; HT: hypertension; MS: multiple sclerosis; SID: systemic inflammatory disease

Clinical events	SLE	RA	PS	IBD	HT	MS
In-hospital mortality, N (%)	30 (12.7%)	45 (12.0%)	20 (9.8%)	20 (8.0%)	0%	2 (14.3%)
Ventricular arrhythmias, N (%)	10 (4.2%)	75 (20.0%)	10 (4.9%)	20 (8.0%)	5 (4.2%)	20 (14.3%)
Cardiogenic shock, N (%)	36 (13%)	35 (9.3%)	15 (7.3%)	30 (12%)	24 (20%)	25 (18%)
Acute heart failure, N (%)	66 (28%)	124 (33%)	64 (31%)	45 (18%)	40 (33%)	35 (25%)
Acute ischemic stroke, N (%)	7 (2.9%)	6 (1.7%)	7 (3.2%)	0%	0%	14 (9.9%)
Complete heart block, N (%)	0%	15 (4.0%)	5 (2.4%)	5 (2.0%)	5 (4.2%)	5 (3.6%)
Acute respiratory failure, N (%)	65 (27.6%)	105 (27.9%)	40 (19.5%)	80 (32.0%)	15 (12.5%)	60 (42.9%)
Acute kidney injury, N (%)	70 (29.8%)	110 (29.3%)	65 (31.7%)	65 (26.0%)	30 (25.0%)	60 (42.9%)
Cardiac arrest, N (%)	10 (4.2%)	15 (4%)	10 (4.8%)	10 (4.0%)	5 (4.1%)	14 (10%)
Mechanical circulatory support, N (%)	5 (2.1%)	35 (9.3%)	5 (2.4%)	10 (4.0%)	5 (4.1%)	5 (3.6%)
Length of stay in days (mean)	5.6	8.3	7.0	12.0	11	10
Total hospital charge in $ (mean)	79374.4	149863.4	132461.6	211938.3	171750.6	171750.6

Univariate analysis comparing those with and without systemic inflammatory diseases (SIDs) revealed that most clinical outcomes did not reach statistical significance (Table 4). However, a few specific disease-event associations stood out.

**Table 4:** Univariate analysis outcomes compared to patients without SID SLE: systemic lupus erythematosus; RA: rheumatoid arthritis; PS: psoriasis; IBD: inflammatory bowel disease; HT: hypothyroidism; MS: multiple sclerosis; CVA: cerebrovascular accident; PCI: percutaneous coronary intervention; MACE: major adverse cardiac events; OR: odds ratio; SID: systemic inflammatory diseases Unadjusted odds ratio of the clinical events amongst the SID cohort compared to the no-SID. *P-value <0.05 considered significant

Clinical events	SLE		RA		PS		IBD		HT		MS	
-	Unadjusted OR (CI)	P-value	Unadjusted OR (CI)	P-value	Unadjusted OR (CI)	P-value	Unadjusted OR (CI)	P-value	Unadjusted OR (CI)	P-value	Unadjusted OR (CI)	P-value
In-hospital mortality	1.3 (0.55-3.07)	0.54	1.2 (0.59-2.45)	0.59	0.96 (0.34-2.69)	0.94	0.77 (0.27-2.16)	0.62	1	-	1.48 (0.51-4.28)	0.46
Ventricular arrhythmia	0.49 (0.11-2.06)	0.33	1.41 (0.6 -2.89)	0.33	0.54 (0.12-2.28)	0.4	0.85 (0.30-2.40)	0.76	0.52 (0.06-3.98)	0.53	0.87 (0.201-3.79)	0.85
Complete heart block	1	-	1.98 (0.62-6.27)	0.24	1.1 (0.16-8.60)	0.86	0.97 (0.13-7.01)	0.97	2.06 (0.27-15.42)	0.47	1.76 (0.23-13.07)	0.58
Acute kidney injury	0.92 (0.49-1.73)	0.80	0.9 (0.55-1.48)	0.69	1.01 (0.52-1.96)	0.96	0.76 (0.41-1.42)	0.4	0.72 (0.28-1.83)	0.49	1.6 (0.77-3.46)	0.19
Cardiac arrest	1.0 (0.24-4.16)	0.9	0.94 (0.3-2.9)	0.92	1.16 (0.28-4.83)	0.85	0.94 (0.22-3.91)	0.93	0.98 (0.13-7.35)	0.99	2.7 (0.81-9.08)	0.1
Cardiogenic shock	0.89 (0.37-2.1)	0.79	0.62 (0.29-1.35)	0.23	0.48 (0.14-1.55)	0.2	0.83 (0.35-1.91)	0.6	1.6 (0.59-4.31)	0.3	1.32 (0.5-3.4)	0.5
Ischemic CVA	3.2 (0.43-24.9)	0.25	1.7 (0.38-8.4)	0.45	9.97 (1.41-70.23)	0.02*	1	-	1	-	1	-
Acute myocardial infarction	0.67 (0.2-2.18)	0.51	1.02 (0.46-2.24)	0.96	0.24 (0.34-1.80)	0.16	0.86 (0.3-2.4)	0.77	0.9 (0.21-3.84)	0.88	3.3 (1.3-7.79)	0.006*
PCI	1	-	3.84 (1.18-12.49)	0.025*	1	-	1.88 (0.25-13.85)	0.53	1	-	3.41 (0.45-25.45)	0.23
Mechanical circulatory support	0.41 (0.56-3.01)	0.38	1.96 (0.90-4.24)	0.08	0.47 (0.06-3.44)	0.46	0.79 (0.19-3.28)	0.75	0.82 (0.19-3.28)	0.75	0.70 (0.95-5.21)	0.73
Acute heart failure	0.98 (0.51-1.86)	0.96	1.28 (0.8-2.06)	0.29	1.1 (0.61-2.31)	0.59	0.56 (0.27-1.14)	0.11	1.28 (0.54-3.01)	0.56	0.85 (0.36-2.01)	0.72
Length of stay	1	-	1	-	1	-	0.46 (0.11-1.88)	0.28	1	-	1	-
MACE	0.92 (0.51-1.66)	0.8	1.65 (1.04-2.6)	0.031*	0.97 (0.52-1.8)	0.92	0.43 (0.22-0.81)	0.01*	1.15 (0.51-2.59)	0.72	1.36 (0.65-2.87)	0.4

Ischemic stroke was significantly more likely in patients with psoriasis (p=0.02); acute myocardial infarction was significantly associated with MS (p=0.006); Percutaneous coronary intervention was significantly more common in RA (p=0.025); Major adverse cardiovascular events (MACE) showed a significantly increased association with RA (p=0.031), while patients with IBD demonstrated a significantly lower likelihood of MACE (p=0.01).

Using Table 5, Multivariate analysis comparing patients with and without systemic inflammatory diseases revealed that after adjusting for key confounders, most clinical outcomes did not achieve statistical significance. However, some disease-specific associations were observed.

**Table 5:** Multivariate analysis of clinical outcomes compared to patients without SID SLE: systemic lupus erythematosus; RA: rheumatoid arthritis; PS: psoriasis; IBD: inflammatory bowel disease; HT: hypothyroidism; MS: multiple sclerosis; CVA: cerebrovascular accident; PCI: percutaneous coronary intervention; MACE: major adverse cardiac events; OR: odds ratio; SID: systemic inflammatory diseases Odds ratio adjusted for sex, race, hyperlipidemia (HLD), coronary artery disease (CAD), hypertension (HTN), peripheral artery disease (PAD), smoking status, diabetes mellitus (DM), chronic kidney disease (CKD), and obesity. *P-value <0.05 considered significant

Clinical events	SLE		RA		PS		IBD		HT		MS	
-	Adjusted OR (CI)	P-value	Adjusted OR (CI)	P-value	Adjusted OR (CI)	P-value	Adjusted OR (CI)	P-value	Adjusted OR (CI)	P-value	Adjusted OR (CI)	P-value
In-hospital mortality	2.16 (0.92-5.04)	0.07	1.02 (0.45-2.28)	0.95	1.34 (0.52-3.46)	0.53	1.02 (0.27-3.89)	0.96	1	-	1.33 (0.47-3.74)	0.585
Ventricular arrhythmia	0.45 (0.1-2.03)	0.3	1.48 (0.7-3.12)	0.3	0.47 (0.11-1.92)	0.29	0.78 (0.26- 2.31)	0.65	0.33 (0.39-2.92)	0.32	0.95 (0.21-4.27)	0.95
Complete heart block	1	-	2.45 (0.70-8.54)	0.15	1.31 (0.14-11.85)	0.80	0.89 (0.1-7.75)	0.92	2.48 (0.28-21.43)	0.40	1.66 (0.14-18.61)	0.68
Acute kidney injury	1.15 (0.61-2.16)	0.64	0.74 (0.38-1.44)	0.38	0.96 (0.46-2.0)	0.92	0.71 (0.28-1.81)	0.47	0.9 (0.33-2.93)	0.94	2.1 (0.65-6.72)	0.21
Cardiac arrest	1.2 (0.27-5.19)	0.8	0.87 (0.27-2.82)	0.82	1.1 (0.25-4.87)	0.88	1.1 (0.3-4.15)	0.8	1.2 (0.15-9.76)	0.85	3.03 (0.78-11.7)	0.1
Cardiogenic shock	0.7 (0.26-2.28)	0.6	0.6 (0.26-1.42)	0.25	0.3 (0.89-1.03)	0.04*	0.69 (0.29-1.63)	0.4	1.58 (0.53-4.71)	0.4	1.15 (0.3-4.21)	0.82
Ischemic CVA	3.2 (0.43-24.9)	0.25	1.79 (0.38-8.4)	0.46	9.9 (1.41-70.23)	0.021*	1	-	1	-	1	-
Acute myocardial infarction	0.49 (0.15-1.54)	0.22	0.63 (0.28-1.4)	0.26	0.26 (0.03-1.83)	0.18	0.92 (0.29-2.87)	0.89	1.16 (0.28-4.79)	0.83	3.06 (1.19-7.84)	0.02*
PCI	1	-	3.71 (1.1-12.3)	0.032*	1	-	2.07 (0.25-17.2)	0.49	1	-	3.8 (0.46-31.9)	0.21
Mechanical circulatory support	0.35 (0.04-2.64)	0.31	2.8 (1.22-6.55)	0.015*	0.32 (0.05-1.94)	0.216	0.59 (0.16-2.16)	0.431	0.65 (0.7-6.2)	0.71	0.6 (0.1-3.58)	0.08
Acute heart failure	0.72 (3.2-1.63)	0.43	1.35 (0.64-2.85)	0.42	1.78 (0.56-5.63)	0.32	0.43 (0.16-1.16)	0.09	1.11 (0.23-5.31)	0.89	0.46 (0.15-1.41)	0.17
Length of stay	1	-	1	-	1	-	0.21 (0.05-0.85)	0.029*	1	-	1	-
MACE	0.69 (0.31-1.51)	0.35	1.41 (0.82-2.43)	0.21	0.96 (0.44-2.09)	0.92	0.32 (0.15- 0.67)	0.003*	1.05 (0.46-2.36)	0.9	0.91 (0.22-3.8)	0.9

Ischemic stroke (CVA), acute myocardial infarction (AMI), percutaneous coronary intervention (PCI), and mechanical circulatory support retained their statistically significant associations. In contrast, patients with inflammatory bowel disease (IBD) had significantly reduced odds of major adverse cardiovascular events (OR = 0.32, p = 0.003) and shorter length of stay (p = 0.029). Cardiogenic shock was significantly less likely in patients with psoriasis (OR = 0.3, 95% CI: 0.89-1.03), though the confidence interval included 1, indicating a null or marginal effect.

## Discussion

This study showed that only 4% of patients hospitalized with AMC had a concomitant SID, yet the patients in this study exhibited profound heterogeneity across demographic, comorbidity, and outcome measures. Patients with RA and MS were older (mean ages 61.5 and 57 years, respectively) than those with SLE (47.5 years) or other SID subtypes, and RA and SLE were female-predominant (80% and 87%), whereas PS, IBD, and non-SID admissions were male-predominant. HT and MS patients were overwhelmingly Caucasian (88% and 80%) compared with a more mixed non-SID population (59% Caucasian, 18% Black, 15% Asian). Traditional cardiovascular risk factors clustered differently across groups, as smoking was highest in RA (36%) and MS (32%), hyperlipidemia and prior myocardial infarction were most common in RA and PS, diabetes burden was highest in MS (21%) and SLE (9%), and chronic kidney disease and obesity varied widely, compounding baseline risk across SID subtypes.

Unadjusted outcomes revealed that, compared to non-SID admissions, SID patients experienced slightly lower in-hospital mortality (8.2% vs. 8.9%) and ventricular arrhythmias (9.6% vs. 11.2%), but higher rates of cardiogenic shock (13.9% vs. 12.9%) and acute heart failure (31.3% vs. 27.5%), longer hospital stays (8.6 vs. 7.6 days), and marginally lower charges ($139,000 vs. $148,000). Among SID subgroups, RA demonstrated the highest crude rates of arrhythmias (20%), mechanical circulatory support (9.3%), and PCI, while SLE had the greatest mortality (12.7%), AKI (29.8%), and respiratory failure (23.4%). MS patients faced extreme respiratory failure (42.9%), AKI (42.9%), and mortality (14.3%), PS had notable AKI (31.7%), and IBD experienced the lowest heart failure rate (18%) but the longest stays (12 days).

On univariate analysis, IBD was linked to reduced odds of acute heart failure and major adverse cardiac events (MACE) (OR 0.43; p = 0.01), RA demonstrated toward higher odds of PCI (OR 3.84; p = 0.025) and MACE (OR 1.65; p = 0.031), PS carried nearly ten-fold increased stroke risk (OR 9.97; p = 0.02), and MS had over three-fold increased AMI odds (OR 3.30; p = 0.006). After adjustment for age, sex, race, smoking, hyperlipidemia, coronary artery disease, hypertension, peripheral arterial disease, diabetes, CKD, and obesity, RA’s odds of mechanical support (aOR 2.8; p = 0.015) and PCI (aOR 3.71; p = 0.032) remained elevated, IBD demonstrated some lower odds of MACE (aOR 0.32; p = 0.003) and prolonged stays (aOR 0.21; p = 0.029), PS continued to show dramatically higher stroke risk (aOR 9.90; p = 0.02), and MS sustained increased AMI risk (aOR 3.06; p = 0.02). SLE and HT did not exhibit statistically significant adjusted associations with major outcomes, despite higher crude rates of acute kidney injury and mortality in SLE (12.7%) and low event counts in HT (0% mortality). The lack of significance after adjustment suggests that comorbidities, other than AMC, drive risk in these populations, or that sample sizes limited statistical power.

RA patients in this study exhibited high rates of hyperlipidemia and active smoking, which are both well‐recognized drivers of endothelial dysfunction and microvascular ischemia. Hence, these cofounders may likely potentiate myocardial microvascular injury in the setting of acute myocarditis and explain the increased incidence of cardiogenic shock and need for percutaneous coronary intervention (PCI) observed in this subgroup. In contrast, IBD patients, many of whom receive anti-tumor necrosis factor (TNF) or other biologic therapies, appeared to benefit from attenuated myocardial inflammation, a phenomenon supported by studies showing that anti‐TNF agents reduce cardiac inflammatory infiltrates and improve ventricular function in experimental myocarditis, thereby translating into fewer heart failure events despite longer hospital stays for close monitoring or treatment optimization. Finally, the markedly elevated risks of ischemic stroke in PS and AMI in MS patients underscore a hypercoagulable state compounded by systemic endothelial activation. PS patients are known to have up to a 50% greater risk of cardiovascular events, likely driven by chronic systemic inflammation and plaque instability, while MS has been linked to abnormal platelet activation and coagulation cascade derangements that predispose to both arterial and venous thromboses, thereby exacerbating ischemic complications in myocarditis [27,28].

In the retrospective cohort, RA patients hospitalized with acute myocarditis had significantly elevated risks of mechanical circulatory support (aOR 2.8; p = 0.015) and PCI (aOR 3.71; p = 0.032), reflecting a burden of myocardial dysfunction likely potentiated by high rates of hyperlipidemia (49%) and smoking (36%). These findings are consistent with the report from a study in Morocco, which described a 56-year-old patient with a six-year history of RA who developed myocarditis confirmed by cardiac MRI [29]. The patient’s favorable response to immunosuppressive therapy further supports the role of immune-mediated injury in RA-associated myocarditis and suggests that, beyond ischemia, active inflammation may contribute directly to myocardial dysfunction in these patients [29].

SLE patients in the retrospective study exhibited the highest crude rates of mortality (12.7%), AKI (29.8%), and respiratory failure (23.4%). However, these associations lost statistical significance after multivariate adjustment, indicating that comorbid burden may drive much of the observed risk.

In a study by Mohanty & Sunder, a 40-year-old SLE patient presented with fulminant heart failure and multi-organ dysfunction, which required intensive care support [24]. After initiation of corticosteroids and mycophenolate mofetil, the patient achieved full recovery of left ventricular function (LVEF improved from 36% to 70%) [24]. This highlights the importance of early recognition and immunosuppressive management in lupus, as it can very much lead to myocarditis. This clinical scenario is similar to the pattern observed in the national cohort, where, despite high crude complication rates, SLE patients demonstrated reversibility with timely therapy.

For psoriasis patients, the retrospective study revealed dramatically elevated risks of ischemic stroke (aOR 9.90; p = 0.02), suggesting a pronounced thromboembolic vulnerability during myocarditis hospitalization. This finding aligns with observations from a study of 100 psoriasis patients, where 5% of screened participants demonstrated active IL-17A-positive myocarditis with elevated TLR-4 expression and anti-heart autoantibodies [25]. Treatment with immunosuppressive agents or IL-17A blockade led to full recovery of left ventricular function, supporting a link between IL-17A-driven systemic inflammation and myocardial injury [25]. The relationship between systemic inflammation, endothelial dysfunction, and hypercoagulability in psoriasis patients may therefore account for the markedly elevated stroke risk seen in the present retrospective study.

The retrospective cohort demonstrated that IBD patients, despite prolonged hospital stays (mean 12 days), experienced a lower risk of major adverse cardiac events (MACE) (aOR 0.32; p = 0.003) and heart failure events. In contrast, a nationwide Swedish study reported that IBD patients faced a 1.55-fold higher long-term risk of myocarditis and a 2.44-fold increased risk of severe myocarditis [30]. This apparent discrepancy may reflect differences between short-term in-hospital and long-term outcomes. The favorable short-term outcomes observed in the national cohort may be explained by the protective effect of biologic therapies commonly used in IBD, such as anti-TNF agents, which may alleviate acute myocardial inflammation during hospitalization while not fully eliminating long-term susceptibility.

These findings highlight that RA, PS, and MS patients require vigilant hemodynamic and thrombotic risk management, potentially including early invasive monitoring and antithrombotic prophylaxis. The unexpected cardioprotective signal in IBD suggests a beneficial role for biologic immunotherapies in attenuating myocardial inflammation. The lack of significant adjusted risk in SLE and HT points to the importance of aggressively managing comorbidities such as CKD, diabetes, and hyperlipidemia in these patients, as these factors appear to drive adverse outcomes more than myocarditis alone.

The use of a large, nationally representative inpatient database and comprehensive adjustment for confounders such as age, sex, comorbidities, and hospitalization characteristics contributes to the strength of this study. The limitations of this retrospective study include potential misdiagnosis and misclassification, lack of outpatient and long-term follow-up data, and small subgroup sizes. Moreover, unmeasured variables such as ejection fraction, biomarker levels, and specific immunosuppressive regimens may further explain observed differences. These limitations constrain the precision of effect estimates, hindering definitive conclusions. However, the findings of this study raise salient points that would inform and benefit further study. Prospective, multidisciplinary studies incorporating angiographic, biomarker, and medication data are needed to validate these associations and to determine whether targeted immunomodulation can improve outcomes in SID-associated acute myocarditis. Randomized trials should evaluate whether tailored immunomodulatory strategies, including early anti-TNF or anti-IL-6 therapies in IBD and RA, can reduce cardiogenic complications and improve functional recovery, building on pilot data in animal models.

## Conclusions

We observed that RA patients had two‐ to three‐fold higher needs for mechanical circulatory support and PCI, whereas inflammatory bowel disease patients experienced fewer heart failure events despite longer hospitalizations, and PS and MS cohorts faced markedly elevated risks of ischemic stroke and AMI, with SLE and HT outcomes driven predominantly by comorbid burden. These subtype‐specific risk signatures highlight the complex interplay between chronic inflammation, endothelial dysfunction, and traditional cardiovascular risk factors in AMC. These findings warrant prospective validation and support the development of tailored clinical guidelines, such as early hemodynamic monitoring and circulatory support for RA, uninterrupted biologic therapy in IBD, antithrombotic prophylaxis in PS and MS, and aggressive management of diabetes, CKD, and hyperlipidemia in SLE and HT, to optimize myocarditis management and improve patient outcomes.
